# Illict drug use and academia in North Kosovo: Prevalence, patterns, predictors and health-related quality of life

**DOI:** 10.1371/journal.pone.0199921

**Published:** 2018-07-16

**Authors:** Tatjana Gazibara, Marija Milic, Milan Parlic, Jasmina Stevanovic, Dragoslav Lazic, Gorica Maric, Darija Kisic-Tepavcevic, Tatjana Pekmezovic

**Affiliations:** 1 Institute of Epidemiology, Faculty of Medicine, University of Belgrade, Belgrade, Serbia; 2 Department of Epidemiology, Faculty of Medicine, University of Pristina temporarily settled in Kosovska Mitrovica, Kosovska Mitrovica, Kosovo, Serbia; 3 Faculty of Dentistry, University of Pristina temporarily settled in Kosovska Mitrovica, Kosovska Mitrovica, Kosovo, Serbia; China Medical University, CHINA

## Abstract

**Purpose:**

The purpose of this study were to estimate the prevalence and patterns of illicit drug use in a sample of University students from North Kosovo, to assess factors associated with illicit drug use and to assess health-related quality of life (HRQoL) among students according to illicit drug use.

**Methods:**

A cross-sectional study was conducted at the Student Public Health Center, where 514 University students were enrolled from April to June 2015 in North Kosovo. Participants completed the general socio-demographic and behavioral questionnaire, Beck Depression Inventory (BDI) and the SF-36 questionnaire for HRQoL assessment. Data on lifetime illicit drug use were self-reported.

**Results:**

As much as 16.0% of students reported ever illicit drug use. The most frequently used drugs were marijuana (9.3%) and bromazepam (7.6%). Factors associated with ever illicit drug use were: being smoker and alcohol user, having chronic diseases and having higher depressive symptoms score. Ever illicit drug users reported all domains of HRQoL as worse.

**Conclusion:**

These results could serve as a tool for implementation of preventive strategies and University policies to promote healthy lifestyles and behaviors. Measurement of HRQoL could also be used as indicator of the effect of interventions designed to reduce and/or prevent illicit drug use at institutions of higher education.

## Introduction

Illicit drug use is defined as use of any chemical substance that has been prohibited by international drug control treaties [1, 2]. These substances inherently alter physical, mental, emotional and/or behavioral functioning of an individual. Major illicit drugs include amphetamine-type stimulants, cannabis (marijuana, hashish), cocaine and opioids (heroin, morphine, methadone and fentanyl) [1]. Worldwide, studies have shown that marijuana is the most common [3, 4] or one of the most commonly used [5–7] illicit drugs among college/University students. Estimates suggest that 8.5% of students initiate marijuana use in the first year of college in the United States (US) (3). Aside from marijuana, use of other illicit drugs such as ecstasy, amphetamines, cocaine, hallucinogens, opiates, inhalants and steroids have been reported during college/University schooling [4–7]. Overall, approximately 21% of college students were identified to have either used illicit drugs, inappropriately used prescription drugs or diverted prescription drugs [8]. Initiation of substance abuse, including illicit drug use, has been considered as factor associated with transition from high school to University, as possibilities for social interactions increase, while young people accept peer norms [9]. Although use of illicit drugs is less common among college students than among young people of same age not attending college [10], illicit drug use is of major concern in this population group.

Several factors, such as depression, anxiety, chronic diseases, perceived stress at University, have been associated with poorer health-related quality of life (HRQoL) among University students [11–13]. Also, it has been observed that excessive alcohol consumption was associated with worse HRQoL in University students [14]. Lev-Ran et al. [15] observed that in a general population aged 18–24 years cannabis use has a strong negative impact on mental health component of the HRQoL. However, there is a lack of studies exploring HRQoL among University students who use illicit drugs and those who do not.

Kosovo currently represents a complex socio-political setting due to ethnic conflict between Albanians and Serbs, resulting in civil war in 1999. Subsequently, Kosovo was administered by the United Nations Mission in Kosovo (UNMIK, UN Security Council Resolution 1244) [16]. Serbs in Kosovo are presently being considered as ethnic minority as most Serbs fled to the north of Kosovo (4 municipalities: Kosovska Mitrovica, Zvečan, Zubin Potok, Leposavić), while a small proportion of Serbs remained in several enclaves (settlements) in southern Kosovo. Administration and governance from the Republic of Serbia is ongoing in North Kosovo, while proper Kosovo Government is not operational in this region. In Serbian enclaves this is not the case [17]. Due to divergence of legislature between the government of Serbia and the government of Kosovo, there are apparent gaps in trade surveillance and inspection of merchandise. As a result, we hypothesized that students in North Kosovo might have more liberal access to illicit substances.

Bearing in mind all above mentioned, the aims of this study were: 1) to estimate the prevalence and patterns of illicit drug use among Serbian students in North Kosovo; 2) to assess factors associated with illicit drug use and 3) to assess HRQoL among students according to illicit drug use history.

## Materials and methods

### Participants and setting

Study participants were undergraduate students registered at the University of Pristina temporarily settled in Kosovska Mitrovica. Pristina is the capital city of the Kosovo province [18]. After the conflict in 1999, The University of Pristina was relocated to Serbia (from 1999 to 2001) and in 2003 was moved to Kosovska Mitrovica (North Kosovo) where it is temporarily settled to date [18]. Presently, the river Ibar geographically divides the city of Kosovska Mitrovica in two parts: smaller, northern part, with predominantly Serbian population (approximately 23,000 inhabitants), and larger, southern part, with predominantly Albanian population (approximately 50,000 inhabitants). The University is located in the northern part of the city. The classes are held in Serbian language.

Students at the University originate from North Kosovo, Serbian enclaves as well as from the Republic of Serbia. The University is consisted of 10 faculties divided in four branches: social sciences and humanities, medical sciences, nature sciences and mathematics, and technology and engineering sciences and presently a total of 8,004 students are enrolled [18]. Participants were recruited between April and June 2015 at the only Student Health Care Center in Kosovska Mitrovica. As regular annual health check-ups are mandatory for all students at the University, this primary-health care facility was convenient for selection of a representative sample of University student population. Sampling was based on convenience. A total of 514 students were included in the study. This sample represents approximately 6.4% of all students of the University of Pristina temporarily settled in Kosovska Mitrovica. Participation was voluntary and anonymous. The approval to conduct the study was obtained from the Ethics Committee of the Faculty of Medicine, University of Pristina temporarily settled in Kosovska Mitrovica.

In the Republic of Serbia, and as well at the province of Kosovo and Metohija which is a constituent part of the territory of the Republic, according to the Constitution of the Republic of Serbia, health care system is mainly financed by mandatory contributions to a social health insurance scheme. Delivery of health care is set according to three levels: primary, secondary and tertiary. Student Public Health Center is the principal primary health care institution for students the University of Pristina temporarily settled in Kosovska Mitrovica, comprising outpatient departments. Moreover, specialist consultations in all clinical fields are provided.

### Instrument

Data were collected by questionnaires. The general questionnaire (S1 Appendix) was related to 1) demographic data: age, gender, place of birth (rural/urban), region of origin (Central Serbia/North of Kosovo/Enclaves), type of faculty (social sciences and humanities/medical sciences/ nature sciences and mathematics/technology and engineering sciences), type of current residence (with parents/student dormitory/rented apartment/other), parental education level, household monthly income; 2) behavior and habits: alcohol use (yes/no) (S2 Appendix), cigarette smoking (yes/no); physical activity (yes/no)–defined as moderate activities for at least 10 min at a time, such as brisk walking, cycling, swimming, or any other activity that causes some increase in breathing or heart rate.

Illicit drugs were considered drugs whose non-medical use has been prohibited under international drug control treaties [2]. These include the plant-based drugs heroin, cocaine, and cannabis, synthetic drugs such as amphetamines, and pharmaceutical drugs such as opioids and benzodiazepines [19]. Illicit drug use was assessed by asking the students to encircle whether or not (yes/no) they have ever used the following drugs listed in the questionnaire: tramadol, methadone, marijuana, hashish, ecstasy, LSD, cocaine and heroin (S3 Appendix). The choice of illicit drugs was made based on types of drugs highlighted in the national study "Health of Population of Serbia" [20].

The Beck Depression Inventory (BDI) was used to explore feelings and attitudes related to general depressive status [21]. It is a one-dimensional scale consisted of 21 items. Answers were graded on a four-point scale from 0 to 3. The total BDI score was obtained as the sum of ratings for each item. The total BDI score ranged from 0 to 63, with higher values denoting presence of more severe depression symptoms.

The HRQoL was assessed by using the SF-36 questionnaire (Serbian translation) [22]. This questionnaire is consisted of 36 questions divided in eight domains/dimensions: Physical Functioning, Role Physical, Bodily Pain, General Health, Vitality, Social Functioning, Role Emotional and Mental Health. Based on these eight domains two summary scores are made: the Physical Composite Score (comprising the former four domains) and Mental Composite Score (comprising the latter four domains). The total quality of life score represented the mean value of the two composite scores. The score ranged from 0 to 100, with higher values denoting better HRQoL.

### Data analysis

Frequency of ever illicit drug users was expressed as percentage. Differences in normally distributed variable were assessed by using t-test for two independent samples. Differences in variables that were not normally distributed were assessed by using the Mann-Whitney test for two independent samples. Chi square test was used to assess differences in categorical variables. To evaluate difference in the SF-36 scores, we applied ANOVA. To examine factors associated with ever illicit drug use we performed univariate logistic regression analysis. Dependent variable in the model was ever illicit drug use (yes/no). Independent variables (risk factors) were: student’s age, gender, place of birth, region of origin, current residence, household monthly income, having own student’s income, parental education level, type of faculty, grade point average, smoking, alcohol use, physical activity, having chronic diseases and BDI. Similarly, we assessed factors associated with marijuana use in a similar manner (dependent variable was ever marijuana use, while the same independent factors were evaluated as previously described). All variables univariately statistically significant and marginally significant (according to Hosmer and Lemeshaw variables with p<0.250) entered the multiple logistic regression models. Similar method was used to assess factors associated with marijuana use. Probability level of ≤0.05 was considered statistically significant. The SPSS 21.0 statistical software package (SPSS Inc, Chicago, IL, U.S.A.) was used to perform the statistical analysis.

## Results and discussion

Basic demographic characteristics according to use of illicit drugs are shown in Table 1. Ever drug users had several different characteristics compared with non-users: they were more likely males, born in urban areas, living in students’ dormitory or alone (in rented apartment), had higher household monthly income, had own income, had higher maternal education level, were smokers and alcohol users. These students also had more chronic diseases and reported higher BDI. A total of 82 students out of 514 (16.0%) reported use of illicit drugs. Frequency of illicit drug use is given in Table 2. The most frequently used drug was marijuana (9.3%). The least frequently reported drug was methadone (0.2%). Males were more likely to have used marijuana (p = 0.001), hashish (p = 0.009), ecstasy (p = 0.008), amphetamines (p = 0.008), and cocaine (p = 0.001).

**Table 1:** Socio-demographic characteristics of students at the University of Pristina temporarily settled in Kosovska Mitrovica according to ever illicit drug use.

Variable	Illicit drug use		P-value
	Never (N = 432)	Ever (N = 82)	
**Age (years)**	20.8 ± 1.6	20.8± 1.9	0.725
**Gender**			0.052
**• male**	192 (44.4)	46 (56.1)	
**• female**	240 (55.6)	36 (43.9)	
**Place of birth**			0.003
**• urban**	263 (60.9)	64 (78.0)	
**• rural**	169 (39.1)	18 (22.0)	
**Region of origin**			0.518
**• Serbia**	126 (49.0)	27 (56.3)	
**• North of Kosovo**	70 (27.2)	13 (27.1)	
**• Enclave**	61 (23.7)	8 (16.7)	
**• *Missing***	*175 (40*.*5)*	*34 (41*.*4)*	
**Type of current residence**			0.001
**• home (with parents)**	120 (27.8)	20 (24.4)	
**• students’ dormitory**	162 (37.5)	27 (32.9)	
**• alone (in rented apartment)**	143 (33.1)	27 (32.9)	
**• other**	7 (1.6)	8 (9.8)	
**Family monthly income (in Euros)**	515 ± 357	805 ± 811	0.004
**Having own income**			0.001
**• yes**	256 (59.3)	65 (79.3)	
**• no**	176 (40.7)	17 (20.7)	
**Mother’s education level**			0.001
**• higher**	96 (22.2)	34 (41.5)	
**• other**	336 (77.8)	48 (58.5)	
**Father’s education level**			0.280
**• higher**	142 (32.9)	32 (39.0)	
**• other**	290 (67.1)	50 (61.0)	
**Type of Faculty**			0.868
**• social science and humanities**	121 (28.0)	22 (26.8)	
**• medical sciences**	198 (45.8)	40 (48.8)	
**• natural sciences and mathematics**	11 (2.5)	3 (3.7)	
**• technology and engineering science**	102 (23.6)	17 (20.7)	
**Grade point average**	7.8 ± 0.9	7.9 ± 0.7	0.447
**Smoking**			0.001
**• yes**	70 (16.2)	46 (56.1)	
**• no**	362 (83.8)	36 (43.9)	
**Alcohol use**			0.001
**• yes**	281 (65.0)	70 (85.4)	
**• no**	151 (35.0)	12 (14.6)	
**Physical activity**			0.285
**• yes**	347 (80.3)	70 (85.4)	
**• no**	85 (19.7)	12 (14.6)	
**Having chronic diseases**			0.001
**• yes**	34 (7.9)	21 (25.6)	
**• no**	398 (92.1)	61 (74.4)	
**Beck Depression Inventory score**	2.8 ± 4.8	8.8 ± 9.5	0.001

Grade point average ranges from 6 as minimum (lowest passing grade) to 10 as maximum (highest passing grade)

Continuous values are presented as mean ± standard deviation; numbers in brackets denote percentages.

**Table 2:** Prevalence of illicit drug use among students of University of Pristina temporarily settled in Kosovska Mitrovica.

Type of illicit drug	Count (%) N = 514	Males (%) N = 238	Females (%) N = 276	P-value
**Marijuana**	48 (9.3)	33 (13.9)	15 (5.4)	0.001
**Bromazepam**	39 (7.6)	15 (6.3)	20 (7.2)	0.672
**Diazepam**	22 (4.3)	7 (2.9)	15 (5.4)	0.164
**Hashish**	12 (2.3)	10 (4.2)	2 (0.7)	0.009
**Cocaine**	10 (1.9)	10 (4.2)	0 (0.0)	0.001
**Ecstasy**	6 (1.2)	6 (2.5)	0 (0.0)	0.008
**Amphetamines**	6 (1.2)	6 (2.5)	0 (0.0)	0.008
**LSD**	6 (1.2)	5 (2.1)	1 (1.4)	0.067
**Heroin**	3 (0.6)	3 (1.3)	0 (0.0)	0.061
**Tramadol**	2 (0.4)	1 (0.4)	1 (0.4)	0.916
**Methadone**	1 (0.2)	1 (0.4)	0 (0.0)	0.281

Table 3 summarizes the results of univariate and multiple logistic regression analysis of factors associated with ever illicit drug use among students the University of Pristina temporarily settled in Kosovska Mitrovica. All statistically significant and marginally significant variables entered the multiple model. Factors associated with use of illicit drugs among students were: cigarette smoking and drinking alcohol, having chronic diseases and having higher depression score (Table 3). According to a similar multiple model, predictors of marijuana use were: being male, not having own income, being smoker and alcohol user, having chronic diseases and having higher score on BDI (Table 4).

**Table 3:** Factors associated with illicit drug use among students of University of Pristina temporarily settled in Kosovska Mitrovica: Results of univariate and multiple logistic regression analysis.

Variable	Univariate logistic regression analysis			Multiple logistic regression analysis		
	OR	95% CI	P-value	OR	95% CI	P-value
**Age**	1.01	0.87–1.16	0.922			
**Gender** **male vs. female**	0.63	0.39–1.01	0.054	0.54	0.26–1.14	0.107
**Place of birth** **urban vs. rural**	0.44	0.25–0.76	0.004	0.98	0.46–2.08	0.953
**Region of origin** **Serbia vs. North Kosovo and Enclaves**	0.80	0.54–1.19	0.262			
**Current residence** **with parents vs. other**	0.83	0.49–1.45	0.528			
**Household monthly income**	n/a					
**Having own income** **yes vs. no**	2.63	1.49–4.64	0.001	1.01	0.38–2.64	0.991
**Mother’s education level** **higher vs. other**	0.40	0.25–0.66	0.001	0.75	0.34–1.67	0.485
**Father’s education level** **higher vs. other**	0.77	0.47–1.24	0.285			
**Type of faculty** **medical science vs. other**	0.89	0.55–1.42	0.624			
**Grade point average**	1.10	0.79–1.53	0.564			
**Smoking** **yes vs. no**	6.61	3.99–10.96	0.001	3.66	1.77–7.60	0.001
**Alcohol use** **yes vs. no**	3.13	1.65–5.97	0.001	2.88	1.08–7.69	0.035
**Physical activity** **yes vs. no**	1.43	0.74–2.76	0.287			
**Having chronic diseases** **yes vs. no**	4.03	2.20–7.40	0.001	2.96	1.21–7.25	0.017
**Beck Depression Inventory score**	1.13	1.09–1.17	0.001	1.10	1.04–1.16	0.001

OR–Odds Ratio; CI–Confidence Interval

**Table 4:** Factors associated with marijuana use among students of University of Pristina temporarily settled in Kosovska Mitrovica: Results of univariate and multiple logistic regression analysis.

Variable	Univariate logistic regression analysis			Multiple logistic regression analysis		
	OR	95% CI	P-value	OR	95% CI	P-value
**Age**	0.94	0.79–1.11	0.465			
**Gender** **male vs. female**	2.80	1.48–5.30	0.002	3.58	1.61–7.95	0.002
**Place of birth** **urban vs. rural**	1.43	0.74–2.75	0.277			
**Region of origin** **Serbia vs. North Kosovo and Enclaves**	1.06	0.65–1.71	0.807			
**Current residence** **with parents vs. other**	1.97	0.90–4.33	0.089	1.45	0.59–3.57	0.420
**Household monthly income**	n/a					
**Having own income** **yes vs. no**	0.21	0.09–0.51	0.001	0.25	0.08–0.75	0.013
**Mother’s education level** **higher vs. other**	2.31	1.25–4.27	0.007	1.76	0.84–3.69	0.136
**Father’s education level** **higher vs. other**	1.59	0.87–2.91	0.131			
**Type of faculty** **medical science vs. other**	1.12	0.61–2.04	0.710			
**Grade point average**	1.13	0.75–1.70	0.563			
**Smoking** **yes vs. no**	0.12	0.65–0.23	0.001	0.20	0.10–0.43	0.001
**Alcohol use** **yes vs. no**	0.28	0.17–0.67	0.005	0.34	0.12–0.98	0.046
**Physical activity** **yes vs. no**	0.47	0.18–1.22	0.124			
**Having chronic diseases** **yes vs. no**	0.35	0.17–0.74	0.006	0.52	0.18–1.46	0.014
**Beck Depression Inventory score**	0.92	0.88–0.95	0.001	0.94	0.90–0.99	0.015

OR–Odds Ratio; CI–Confidence Interval

The HRQoL scores according to ever illicit use are presented in Table 5. Ever illicit drug users reported all domains of HRQoL as worse. Similarly, these students had worse Physical and Mental Composite Scores as well as the total HRQoL score (Table 5).

**Table 5:** Mean T scores of the SF-36 scales among students of University of Pristina temporarily settled in Kosovska Mitrovica according to illicit drug use.

Scales of SF-36	Illicit drug use		P-value
	Never (N = 432) Mean ± SD	Ever (N = 82) Mean ± SD	
**Physical functioning**	93.5 ± 17.0	89.1 ± 20.7	0.040
**Role physical**	88.6 ± 23.1	72.0 ± 35.4	0.001
**Pain**	87.4 ± 16.1	77.2 ± 23.3	0.001
**General Health**	75.2 ± 16.4	70.4 ± 19.3	0.019
**Vitality**	70.6 ± 18.9	59.0 ± 24.3	0.001
**Social Functioning**	83.1 ± 20.9	71.6 ± 26.2	0.001
**Role Emotional**	83.2 ± 32.5	58.5 ± 40.8	0.001
**Mental Health**	76.4 ± 18.3	62.7 ± 23.5	0.001
**Physical Composite Score**	83.1 ± 12.1	73.5 ± 18.0	0.001
**Mental Composite Score**	77.7 ± 15.7	64.5 ± 20.9	0.001
**Total Score**	82.2 ± 13.5	70.1 ± 19.1	0.001

SD-standard deviation

Consistent with previously reported prevalence [8], the results of our survey provide evidence that one in six students in North Kosovo have ever used illicit drugs. In an attempt to quantify illicit drug use various authors reported substantially different prevalence estimates [3–7, 23]. These differences may be attributable to inclusion of various substances, which may not be illicit *per se* in majority or regions worldwide (e.g. alcohol or tobacco) [5, 6, 23] or are not covered by questionnaire batteries (e.g. steroids, inhalants) [4]. Therefore, a comparison of overall prevalence of illicit drug use may not be adequate. Moreover, Shamsipour et al. [24] suggested that conventional methods of direct questioning by self-report in assessment of illicit drug use may yield quite lower prevalence when compared with a ‘crosswise model’ technique applied several months afterwards. Even though analyzing drug metabolites in biological specimens may be the most accurate method to determine the extent of illicit drug use, their applicability in population-based cross-sectional studies is limited.

In our survey illicit drug users most commonly reported use of marijuana (9.3%). These findings are in accordance with those reported in previous studies [4, 10]. However, prevalence of marijuana use was lower compared with college students in the US for example, where lifetime prevalence of 30% at college entry [3] or 60% among sophomores were observed [4]. In other regions, such as Iran or India, the prevalence of marijuana use of 7.4% and 6.8% respectively, has been estimated [5, 6]. Peer use of marijuana seems to be associated with initiation of marijuana use among non-users [25]. Studies have shown that use of marijuana may predict use of other illicit drugs, such as hallucinogens or ‘hard drugs’ [26], which may have other important public health implications.

Furthermore, misuse of tranquilizers, ranked second among our students, has also been recognized in a college setting [4, 27]. A recent study on prescription drug misuse reported that 25% of college students use these drugs without physician’s prescription [27]. Analysis of motives for prescription drug misuse suggested that most common reasons include self-treatment intentions, recreation or both [28]. At the same time, one half of college students who misuse prescription drugs are screened positive for drug abuse [28]. A small percentage of students in our survey reported the use of ecstasy and amphetamines. Ecstasy and amphetamines are commonly labelled as “club drugs”. Their psychoactive effects include enhanced feelings of closeness with others and sensory perception [10]. Sim et al. [29] observed that those students who recreationally use ecstasy are more likely to initiate illicit drug use at younger age and engage in binge drinking and marijuana use. Amphetamines, on the other hand, could be used with the goal to improve academic performance and concentration [30]. Cocaine use was reported by 1.9% of students in our survey. By contrast, cocaine use was ranked second in terms of both lifetime and past-month use among illicit drug users in a US college [4]. In addition, in the US, 13% of students reported cocaine use by fourth year of college [31]. On the other hand, in a survey among male students in India and among undergraduate students in Ethiopia no use of cocaine, amphetamines, sedatives or heroin was reported [6, 32]. These remarkable variations might be a result of differences in definition of time frame for which illicit drug use was assessed: illicit drug use during the past month [6, 31] versus lifetime prevalence of illicit drug use in our study. Also, these variations could be explained by differences in financial means for purchase, as it has been suggested that the trajectory of cocaine and marijuana use, both in college and in general population, is being influenced by changes in price [33].

We observed that students who smoked cigarettes and drank alcohol were more likely to be illicit drug users. Our results are in line with the existing literature data [3, 5, 6, 33, 34]. In addition, male students were more likely to use marijuana. Male sex, smoking and alcohol consumption have been consistently associated with illicit drug use [3, 5, 6, 33–36]. These factors are likely related to particular behavioral patterns, as studies have shown that male college students are more likely to exert high-risk behaviors [35, 36], which could be a result of gender-role stereotypes and the socialization process [37]. Specifically, Davies et al. suggested that boys are encouraged from early childhood to engage in riskier behaviors that are deemed as display of strength and fearlessness based on stereotyping and traditional notions of masculinity [37], which might explain a higher propensity towards risk-taking among males irrespective of ethnicity, religion or socio-economic background.

In this survey, we also observed that ever illicit drug users were more likely to have chronic diseases and a higher level of depression. It has been estimated that around 30% of University students have chronic diseases and/or special care needs [8]. In management of health conditions, use of illicit substances might complicate the diagnosis and treatment [8]. There are some indices in the available body of literature that high school students with asthma are more likely to use illicit drugs [38], which corresponds to our findings. However, illicit drugs use remains understudied topic in the population of University students with chronic diseases.

We also observed that higher BDI score was associated with illicit drug use in our sample of students. Due to our cross-sectional study design we cannot determine whether being depressed lead to use of illicit drugs or vice versa. Nevertheless, it is important that University students are offered screening for depression as part of the periodical general health status assessment during schooling. In this way, vulnerable individuals could be assessed for illegal drug use also. Subsequently, these students could be offered support and counseling with the goal to reduce, minimize or alter their risky behavior. A study of personal concerns of college students revealed a wide spectrum of negative consequences that college student experience due to drug use [4]. The highest proportions of lifetime negative consequences accounted for having said or done something embarrassing (46%), felt guilty and ashamed (45%), not having done their academic duties or received a lower grade (44%) and feeling bad physically (43%) [4]. Those students who reported ever illicit drug use had worse scores in all HRQoL domains. Similarly, physical and mental composite scores as well as the overall HRQoL were worse in students who have ever used illicit drugs. Studies exploring HRQoL among University students are scarce [39]. Caldeira et al. [39] followed a cohort of 1,253 college students throughout six years, starting from their freshmen year. Over this period, out of six subgroups describing marijuana-use frequency in college students, chronic marijuana users had worse physical and psychological health [39]. Lower HRQoL has also been identified among adolescents who engage in risky behaviors as opposed to those students who do not [40]. To explain this finding, Topolski et al. [40] suggested that factors such as relationship with parents might mediate the relation between illicit drug use and worse HRQoL. This means that adolescents who engaged in risky behaviors were less likely to get along with their parents [40]. Parental monitoring and vigilance concerning their adolescent's activities and friends could be seen as proxy of getting along with parents. Taking this into consideration, a higher level of parental monitoring and supervision during senior year in high school has been associated with having less drug-using peers, which was, in turn, associated with decreased illicit drug exposure in college [25].

Information bias should be taken into account as a limitation of this study, because data on illicit drug use, smoking, alcohol and physical activity were self-reported. Thus, it is possible that data collected in this study were under-reported. The cross-sectional study design precludes us from drawing the direction of inference, and therefore associations could only suggest potential associations and not determine direction of causality.

## Conclusions

In conclusion, this study illustrated illicit drug use patterns in academic environment in North Kosovo. There are several factors that remain consistent with literature in terms of likelihood of illicit drug use such as being male, smoker and drinking alcohol. Information regarding illicit drug use in academic setting could provide orientation and serve as a tool for implementation of preventive strategies and university policies to promote healthy lifestyles and behaviors. Measurement of HRQoL could also be used as indicator of the effect of interventions designed to reduce and/or prevent illicit drug use at institutions of higher education. Future studies should focus on development and implementation of health interventions in academic setting with the goal to provide supportive and safe education environment with long-term benefits for the society as a whole.

## Supporting information

S1 Appendix Socio-demographic questionaire. (DOC)

S2 Appendix Questionnaire on the use of alcoholic beverages. (DOC)

S3 Appendix Questionnaire on the use of psychoactive substances. (DOC)
