# Supplementary material for: Illict drug use and academia in North Kosovo: Prevalence, patterns, predictors and health-related quality of life
Source: PLoS One. 2018 Jul 16;13(7):e0199921. doi: 10.1371/journal.pone.0199921 (PMC6047773; doi:10.1371/journal.pone.0199921)
Supplement: S1 Appendix — (DOC) [file pone.0199921.s001.doc]

**S1 Appendix. Socio-demographic questionaire**

1. **Sex**: a) male b) female

2. **Age** _________________ 3.**Faculty/college:**___________________ 4. **Year of study:** _______ 5. **Grade point average:** __________________

6. **How many times did you repeat a study year (total):**___________________

7. **Place of residence before studying:** a) town b) village

8. **Current place of residence:**

a) with parents b) student dormitory c) rented flat d) other

9. **What are your total monthly earnings (scholarships, money you get from your parents, other):**_______________________________________________________

10. **Your father's education level:**

a) elementary b) vocational training c) secondary d) university

11. **Your mother's education level:**

a) elementary b) vocational training c) secondary d) university

12. **What is the total monthly income of your family:**______________________

13. **Are you :** a) smoker b) non-smoker c) ex-smoker

*Smoker is a person who smokes at least one cigarette per day or has smoked 100 cigarettes in their lifeltime.*

14.**When did you start smoking (age): _________________________**__________

15. **Select your reasons for starting to smoke from the choices below:**

a) curiosity b) feeling of security c) imitation of friend d) imitation of parents e) imitation of brother, sister f) loneliness g) calming of anxiety (tension reduction) h) tobacco smoke is pleasant i) other

16. **How many cigarettes do you smoke (or smoked) on average daily, if you smoke every day:** _____________________________________________________

17. **How many times have you tried to stop smoking:**

a) none b) 1-2 times c) several times

18.**What was the longest discontinuation of smoking:** _____________________________________________________________________

19. **How many years have you been smoking continuously: _____________________________________________**_________

20. **If you are a former smoker, when did you stop smoking (age):** _____________________________________________________________________

21. **Give reasons why you stopped smoking:**

a) smoking is harmful b) cigarettes are expensive c) social inadmissibility d) due to illness e) other _______

22. **Do you stay or have you stayed a the room where others smoke:** a) yes b) no

**If yes, on average how many hours per day:** _____________________________________________________________________

23. **Do you live with:** a) smoker(s) b) non-smoker(s)

24 . **Do you have or have had any of the following:**

a) diabetes b) high blood pressure c) heart failure

d) chronic bronchitis e) bronchial asthma f) neurological diseases (which) ________________ g) mental illness h) gastric and intestinal ulcer i) other _______

25. **How often do you drink coffee:** a) never b) occasionally c) every day

26. **Do you regularly have breakfast:** a) yes b) no

27. **How many portions of fruit you consume during the day:** _______________

*One portion of fruit comprises two small fruits (i.e. 2 plums) or 1 medium sized apple*

28. **How many portions of vegetable you consume during the day:** ____________

*One portion of vegetables comprises two table spoons of raw vegetables or 3 tables spoons of cooked vegetables*

29. **How often do you eat meat:**

a) very rarely b) monthly c) weekly d) 2-3 times a week e) every day

30. **How often do you do sports:**

a) never b) monthly c) weekly d) daily

31. **How often do you do recreation:**

a) rarely b) 2 times a week (20 minutes of exercise, intensive walks, and so on)

c) daily (20 minutes of exercise, intensive walks and so on)

32. **Are you physically active?**

a) yes, I am an active athlete b) yes, I exercise regularly c) occasionally, with friends d) I do not do sports, sometimes i go for a walk

33. **If you are physically active, how often do you practice it?**

a) daily (how much time ______) b) 2 -3 times a week c) once a week d) 2-3 times a month e) once a month

34. **How many hours a day do you spend sitting?**

a) up to 3 hours b) up to 5 hours c) up to 8 hours d) more than 8 hours

35. **How many hours a day do you sit surfing the Internet, playing computer games, chatting, etc.?**

a) 1-2 hours b) up to 5 hours c) more than 5 hours d) I do not use a computer for the stated purposes
