# Supplementary material for: Illict drug use and academia in North Kosovo: Prevalence, patterns, predictors and health-related quality of life
Source: PLoS One. 2018 Jul 16;13(7):e0199921. doi: 10.1371/journal.pone.0199921 (PMC6047773; doi:10.1371/journal.pone.0199921)
Supplement: S2 Appendix — (DOC) [file pone.0199921.s002.doc]

**S2 Appendix. Questionnaire on the use of alcoholic beverages**

1. **How often do you drink alcoholic beverages?**

a. never d. 2-3 times per week

b. monthly e. 4 or more times a week

c. 2-4 times in a month

2. **When you drink alcoholic beverages, how often do you drink in a day:**

a. number of schnapps, brandy, whiskey glasses:

ٱ 1-2; ٱ 3-4; ٱ 5-6; ٱ više od 6

b. number of wine glasses:

ٱ 1-2; ٱ 3-4; ٱ 5-6; ٱ 7-9; ٱ more than 10

c. number of beer glasses:

ٱ 1-2; ٱ 3-4; ٱ 5-6; ٱ 7-9; ٱ more than 10

3. **How often do you drink 6 or more drinks in a row on a single occasion?**

a. never

b. monthly

c. 2-3 times per week

d. 4 or more times per week

4. **How often did you get drunk in the past year?**

a. never

b. monthly

c. 2-3 times per week

d. 4 or more times per week

**5. . How often have you been drunk for the past 30 days?**

a. never

b. monthly or never

c. 2-3 times per week

d. 4 or more times per week
