# Supplementary material for: Illict drug use and academia in North Kosovo: Prevalence, patterns, predictors and health-related quality of life
Source: PLoS One. 2018 Jul 16;13(7):e0199921. doi: 10.1371/journal.pone.0199921 (PMC6047773; doi:10.1371/journal.pone.0199921)
Supplement: S3 Appendix — (DOC) [file pone.0199921.s003.doc]

**S3 Appendix. Questionnaire on the use of psychoactive substances**

1. **Up this point have you used (tried):**

|  |  | How many times in the last:  12 months 30 days | Age at first use |
| --- | --- | --- | --- |
| Bensedin | NO YES, | _________ __________ |  |
| Lexilium | NO YES, | _________ __________ |  |
| Bromazepam | NO YES, | _________ __________ |  |
| Trodon | NO YES, | _________ __________ |  |
| Metadon | NO YES, | _________ __________ |  |

2. **Up this point have you used (tried):**

|  |  | How many times in the last:  12 months 30 days | Age at first use |
| --- | --- | --- | --- |
| Marijuana | NO YES, | _________ __________ |  |
| Hashish | NO YES, | _________ __________ |  |
| Amfetamine (speed) | NO YES, | _________ __________ |  |
| Ecstasy | NO YES, | _________ __________ |  |
| LSD | NO YES, | _________ __________ |  |
| Cocaine | NO YES, | _________ __________ |  |
| Heroin (horse, dope) | NO YES, | _________ __________ |  |

3. **Which of the above mentioned drug did you use first?** ____________________

Why?________________________________________________________________

4. **Have you tried to stop using drugs?** NO YES **How many times**: _______

5. **Do you want to stop taking drugs?** NO YES

6. **Do you approve smoking marijuana?**  NO YES

7. **Do you approve of taking other drugs:** NO YES

8. **Do you think that drug use damages health?** NO YES

9. **Do you drink alcoholic drinks while doing drugs?** NO YES

**If yes, which drink do you consume most often? ____________________________**
